# Training needs assessment for clinicians at antiretroviral therapy clinics: evidence from a national survey in Uganda

**DOI:** 10.1186/1478-4491-7-76

**Published:** 2009-08-23

**Authors:** Ibrahim M Lutalo, Gisela Schneider, Marcia R Weaver, Jessica H Oyugi, Lydia Mpanga Sebuyira, Richard Kaye, Frank Lule, Elizabeth Namagala, W Michael Scheld, Keith PWJ McAdam, Merle A Sande

**Affiliations:** 1 Infectious Diseases Institute, Makerere University, Kampala, Uganda; 2 DIFAEM – German Institute of Medical Mission, Tuebingen, Germany; 3 Department of Global Health and International Training and Education Centre on HIV (I-TECH), University of Washington, Seattle WA, USA; 4 Coordinating Center for Infectious Diseases, Centers for Disease Control and Prevention, Lilongwe, Malawi; 5 African Palliative Care Association, Kampala, Uganda; 6 Regional Office for Africa, World Health Organization, Brazzaville, Congo; 7 Ministry of Health, Kampala, Uganda; 8 Department of Internal Medicine, University of Virginia, Charlottesville VA, USA; 9 Department of Clinical Tropical Medicine, London School of Hygiene and Tropical Medicine, London, UK; 10 Pratt Medical Group, Tufts-New England Medical Center, Boston MA, USA; 11 Formerly of the Department of Medicine, University of Washington, Seattle, WA, and the Accordia Global Health Foundation, Arlington, VA, USA

## Abstract

**Background:**

To increase access to antiretroviral therapy in resource-limited settings, several experts recommend "task shifting" from doctors to clinical officers, nurses and midwives. This study sought to identify task shifting that has already occurred and assess the antiretroviral therapy training needs among clinicians to whom tasks have shifted.

**Methods:**

The Infectious Diseases Institute, in collaboration with the Ugandan Ministry of Health, surveyed health professionals and heads of antiretroviral therapy clinics at a stratified random sample of 44 health facilities accredited to provide this therapy. A sample of 265 doctors, clinical officers, nurses and midwives reported on tasks they performed, previous human immunodeficiency virus training, and self-assessment of knowledge of human immunodeficiency virus and antiretroviral therapy. Heads of the antiretroviral therapy clinics reported on clinic characteristics.

**Results:**

Thirty of 33 doctors (91%), 24 of 40 clinical officers (60%), 16 of 114 nurses (14%) and 13 of 54 midwives (24%) who worked in accredited antiretroviral therapy clinics reported that they prescribed this therapy (p < 0.001). Sixty-four percent of the people who prescribed antiretroviral therapy were not doctors. Among professionals who prescribed it, 76% of doctors, 62% of clinical officers, 62% of nurses and 51% of midwives were trained in initiating patients on antiretroviral therapy (p = 0.457); 73%, 46%, 50% and 23%, respectively, were trained in monitoring patients on the therapy (p = 0.017). Seven percent of doctors, 42% of clinical officers, 35% of nurses and 77% of midwives assessed that their overall knowledge of antiretroviral therapy was lower than good (p = 0.001).

**Conclusion:**

Training initiatives should be an integral part of the support for task shifting and ensure that antiretroviral therapy is used correctly and that toxicity or drug resistance do not reverse accomplishments to date.

## Background

Considerable progress continues towards increasing access to anti-retroviral therapy (ART) in resource-limited settings. WHO, UNAIDS and UNICEF recently estimated that 2.12 million people have access to ART in sub-Saharan Africa, or 30% of people with HIV living there who need ART [1]. These accomplishments required training of health professionals, among other efforts to strengthen health systems. For example, the United States President's Emergency Plan for AIDS Relief supported the training or retraining of 219 700 health professionals in ART from 2004 to 2008 [2].

The greatest challenge to increasing access to ART, however, is the shortage of trained health care professionals [1,3-5]. Several experts recommend "task shifting" from doctors to clinical officers and nurses [6-9] or from clinicians to community health workers [8-10]. According to WHO, task shifting is the rational redistribution of tasks among health workforce teams:

"Specific tasks are moved, where appropriate, from highly qualified health workers to health workers with shorter training and fewer qualifications in order to make more efficient use of available human resources for health." [8]

Gimbel-Sherr et al. demonstrated that expanding the role of nurses allowed doctors to have more visits with ART-eligible patients at two clinics in Mozambique [11]. Last year, they compared ART patients treated by non-physician clinicians to those treated by doctors, and reported that the quality of services provided by non-physician clinicians was equivalent to or slightly better than that of doctors [12]. Recent articles report on clinical officers and/or nurses providing ART in Kenya [13,14], Malawi [15], Rwanda [16] and Zambia [17,18].

In 2008, WHO published global recommendations and guidelines for task shifting that would promote access to HIV and other health care services [8]. Recommendation Four is that countries undertake or update a human resource analysis on the extent to which task shifting is already taking place, among other things. Recommendation Nine is that countries adopt a systematic approach to harmonized, standardized and competence-based training that is needs-driven and accredited. The Infectious Disease Institute (IDI), in collaboration with the Ministry of Health (MOH) of Uganda, recently conducted a training needs assessment that addressed both of these recommendations. Information was collected on the allocation of ART tasks across health professionals. An audience analysis [19] provided background on previous training and self-assessment of HIV and ART knowledge.

Uganda was chosen for its well-developed national ART programme and mature training environment for HIV care. As of September 2007, an estimated 111 232 people had access to ART, or 33% of people in need [1]. Uganda also was a pioneer in training nurses to perform some tasks of doctors, and lay health workers to perform some tasks of nurses [20,21]. Health professionals benefited from several ART training initiatives, including the Drug Access Initiative [20], and WHO's Integrated Management of Adult and Adolescent Illness [21], as well as training from organizations such as the Joint Clinic Research Centre, HealtheFoundation [22], IDI [20,22,23], Mildmay International [20,22], Paediatric Infectious Diseases Clinic at Mulago Hospital, The AIDS Support Organization (TASO) [22], and Uganda Cares. These organizations trained a variety of health professionals with courses lasting from one day to 21 days.

This assessment contributes a method for identifying task shifting that has occurred in resource-limited settings and measuring the ART training needs associated with it to the literature on ART training needs. Previously, the Center for African Family Studies and Regional AIDS Training Network conducted an HIV/AIDS training needs assessment in 12 countries in 2002 that predated scaling-up of ART (Marc Ahmed Okunnu, personal communication, 10 August 2009). Renggli conducted a situational analysis in Africa that focused on organizations that provided training in clinical management of HIV infection, including ART [20]. Souville et al. reported on knowledge of and attitudes about ART among physicians in Cote d'Ivoire [24], and Dohrn et al. reported on knowledge of ART among midwives in South Africa [25]. The innovative method presented below can be replicated to inform ART training programmes in the context of on-going scale-up and shifting tasks.

## Methods

### Study design

We surveyed health professionals and heads of ART clinics at a cross-section sample of clinics that the MOH had accredited to provide ART. Health professionals reported on the tasks they performed during a normal work day, previous HIV training and overall knowledge of HIV and ART. Knowledge was rated on a six-point scale, where one was "excellent" and six was "none." The heads of ART clinics reported on the staff and patients at the HIV and ART clinic.

### Sampling procedure and sample size

We sought a nationally representative sample of accredited ART clinics in Uganda. The Ugandan health system divides the country into 11 catchment areas of the regional referral hospitals. Each area serves several districts. The national referral hospital in Kampala is the twelfth area; it was excluded from the assessment, because IDI sought information to guide training programmes for health professionals outside of Kampala. Using a lottery method, the following six areas were selected: Arua, Lira, Masaka, Hoima, Kabale and Mbale.

Using proportionate allocation to size sampling method, a sample 44 of the 205 accredited facilities as of July 2006 was selected. According to the Ministry of Health, (personal communication, MOH, National Medical Stores, 2006), 12% of the accredited ART clinics in the six catchment areas were regional referral hospitals, 35% were district hospitals, and 54% were health centre IV or III. The sample included six regional referral hospitals (14%), 16 district hospitals (34%), and 22 health centre IV or III (52%).

In each catchment area, a random sample of facilities was selected from a MOH list of accredited ART clinics, stratified by type of facility. The two strata were: ownership (government or nongovernmental organization and/or faith-based organization) and whether or not the facility was active, i.e. providing ART. Three government district hospitals were selected randomly from each of the four catchment areas of the biggest administrative regions and two from each of the others. One facility that was not active was selected from each catchment area. At least one nongovernmental or faith-based facility was selected from each catchment area, including six hospitals and four health centres. Two remote facilities were replaced with proximate ones to stay within the bounds of the study schedule and budget.

Within health facilities, a convenience sample of health professionals was selected with the help of the head of the ART clinic. The inclusion criterion was any person providing services at the accredited ART clinic who was at the facility on the day that the study team visited (see below). We sought to include at least one doctor, clinical officer, nurse and midwife from each clinic. In Uganda, a doctor has secondary school education (13 years), five years of medical school and one year of internship. Clinical officers are among the non-physician clinicians described in a recent review [26]; they have a secondary school education, three years of pre-service training and two years of internship. There are several types of nurses: all have a secondary school education; (1) enrolled nurse and enrolled midwives have one and one-half years of pre-service training; (2) comprehensive nurses, registered nurses and registered midwives have three years of pre-service training; and (3) double-trained nurse-midwives have four and one-half years of pre-service training.

### Data collection procedures

Data were collected by means of self-administered questionnaires for individual health professionals and face-to-face interviews with heads of ART clinics as key informants. The questionnaires were designed based on examples from the National Evaluation Center of the United States AIDS Education and Training Centers. (See ) The questionnaire for individual health professionals had six sections on (1) professional background, (2) provision of HIV/AIDS services, (3) training in HIV/AIDS, (4) barriers to training, (5) attendance at IDI courses, and (6) IDI's AIDS Treatment Information Center. The questionnaire for the head of the ART clinic had similar sections, but only the responses to questions about staff and patients at the HIV and ART clinics were used in the analysis.

Early versions were shared with stakeholders representing HIV training organizations in Uganda in a participatory process that led to several improvements. Later versions of the questionnaires were pretested with health professionals and the head of the ART clinic at Mbuya Reach Out and a Kampala City Council clinic. The final questionnaire for health professionals is included as Additional file 1; the questionnaire for the head of the ART clinic is available from the authors on request.

Twelve research assistants were trained in data collection for three days. They were grouped into four teams, each comprising a social scientist, medical doctor and field assistant. A team spent one day at each facility and collected data from an average of 11 facilities during a two-week period in July and August 2006.

### Data management and analysis

The completed questionnaires were coded and the data were double-entered in Epi-Info version 6.01 software (Centers for Disease Control and Prevention, Atlanta GA, United States of America) to ensure accuracy and integrity of the data. Descriptive statistics and statistical tests were conducted with SPSS-PC software, version 11.0 for Windows (SPSS Inc, Chicago IL, United States of America). Data analyses were stratified by health profession and chi-square (χ^2^) tests were used to assess statistical significance of differences in proportions (percentages). Where there were a small number of cases (expected frequency less than 5); Fisher's exact tests were used.

### Human subjects

The study was approved by the IDI training committee, the MOH and the Institutional Review Board of the Faculty of Medicine at Makerere University. Respondents provided oral informed consent.

## Results

### Characteristics of the sample

**Figure 1:**
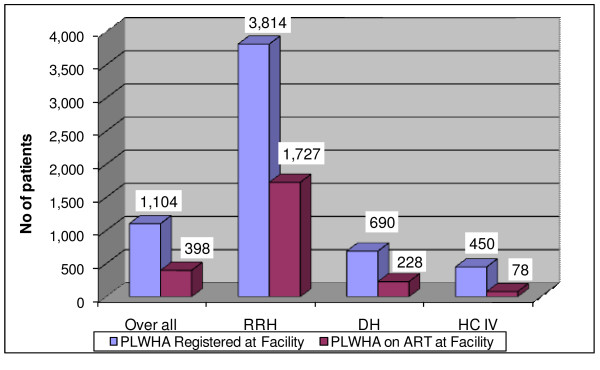
**Average number of registered people living with HIV/AIDS, and ART patients in the facility**.

Forty-three of the 44 facilities selected were included; a team was unable to travel to one nongovernmental health centre that was not active. Thirty-eight of the 43 health facilities were active and five (one district hospital and four health centre IVs) were not. As shown in Figure 1, the regional referral hospitals provided ART to an average of 1727 HIV patients per month, whereas the district hospitals and health centre IV provided ART to an average of 228 and 78 people, respectively. Regional referral hospitals reported the highest proportion of HIV patients receiving ART (45%), while 33% and 17% of HIV patients received ART at district hospitals and health centre IVs, respectively.

The sample of health professionals included 265 clinicians: 34 were doctors, 46 clinical officers, 124 nurses and 61 midwives. Sixty percent were female and 58% were aged 35 years or younger. Table 1 compares the respondents to the staff that the head of the ART clinics reported were assigned to the ART clinics. The distribution of respondents across health professions differed significantly from the distribution of staff assigned to the ART clinics. Doctors were underrepresented at all types of facilities; nurses were underrepresented at regional referral hospitals and district hospitals and overrepresented at health centre IVs. No doctors were on the staff of ART clinics at two district hospitals and two health centres.

**Table 1:** Comparison of respondents and all health professionals in the sample of ART clinics

**Cadre**	**Total**		**Regional referral hospital**		**District hospital**		**Health centre IVs**	
	
	Respondents (n = 265) %	Staff reported by head of clinic (n = 392) %	Respondents (n = 54) %	Staff reported by head of clinic (n = 81) %	Respondents (n = 101) %	Staff reported by head of clinic (n = 158) %	Respondents (n = 110) %	Staff reported by head of clinic (n = 153) %
Doctor	13	19	22	28	14	20	7	14

Clinical Officer	17	21	11	19	17	18	21	26

Nurse	47	44	41	48	47	53	50	33

Midwife	23	15	26	5	23	9	22	27

p-value	0.014		0.005		0.017		0.033	

### Allocation of tasks by health profession

ART tasks were performed by all types of clinicians, as shown in Table 2. Thirty of 33 doctors (91%), 24 of 40 clinical officers (60%), 16 of 114 nurses (14%) and 13 of 54 midwives (24%) who worked in accredited ART clinics reported that they prescribed ART (p < 0.001). Of the 83 people who prescribed ART, only 36% were doctors; 29% were clinical officers, 19% nurses and 16% midwives. On a normal working day, nurses reported spending more than the average number of hours prescribing ART and midwives reported spending less. Consequently, of the 234 hours devoted to prescribing ART, 36% were by doctors, 30% by clinical officers, 24% by nurses and 9% by midwives.

**Table 2:** Allocation of tasks in ART clinics by profession

	**Doctor**	**Clinical Officer**	**Nurse**	**Midwife**			
	
**Tasks**	n = 33	n = 40	n = 114	n = 54	Bivariate analysis		
	
	%	%	%	%	χ^2^	dF	*p*-value
Clinical care							

Prescribing ART	90.9	60	14	24.1	82.89	3	<0.001*

Prescribing other medicines	100	90	43.9	57.4	42.13	3	<0.001*

Providing basic HIV care	69.7	70	58.8	50	6.04	3	0.152

Nursing care and counselling							

Counselling clients	54.5	52.5	71.9	90.7	24.08	3	<0.001*

Nursing care to PLWHA	15.2	15	53.5	48.1	32.72	3	<0.001*

Home visiting	12.1	20	35.1	37	10.3	3	0.022*

Other aspects							

Administration/Supervisor	78.8	40	35.1	46.3	22.54	3	<0.001*

Training other health professionals	54.5	15	9.6	29.6	34.37	3	<0.001*

Health education	48.5	60	70.2	96.3	28.4	3	<0.001*

Others	9.1	10	20.2	13	7.28	3	0.245

*Significantly different

### HIV training

Eighty-six percent of respondents reported they had attended at least one HIV training session, and percentages did not differ significantly across health professions (p = 0.242). The median duration of training varied across topics from three days for infection control to 21 days for HIV research. The median duration of training in initiating ART was seven days, and also seven days in monitoring ART.

The percentage of people with training in specific topics differed significantly across health professions, as shown in Additional file 2. Seventy-one percent of doctors and 54% of clinical officers attended training on initiating ART, compared to 54% of nurses and 40% of midwives (p < 0.001). Higher percentages of doctors and clinical officers attended training on monitoring ART (p = 0.001) and paediatric HIV care (p = 0.023) than nurses and midwives. Conversely, lower percentages of doctors and clinical officers attended training on voluntary counselling and testing (p = 0.003) than nurses and midwives.

Focusing on ART training among respondents who reported that they prescribed ART, 24% of doctors, 38% of clinical officers, 38% of nurses and 49% of midwives had no training in initiating patients on ART (p = 0.457). Twenty-seven percent of doctors, 54% of clinical officers, 50% of nurses and 77% of midwives had no training in monitoring patients on ART (p = 0.017).

### Self assessment of HIV and ART knowledge

**Figure 2:**
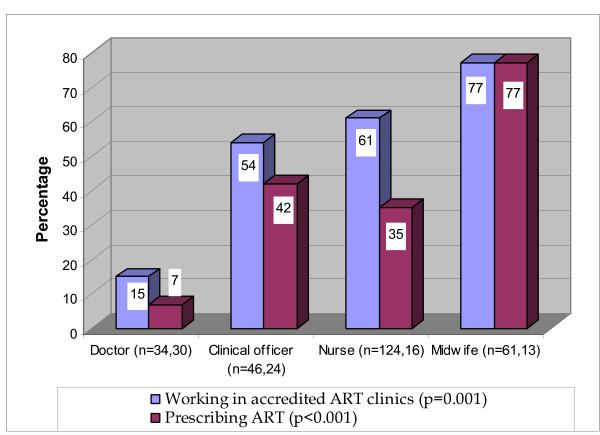
**Percentage of health professionals who assessed their overall knowledge of ART as less than "good"**.

Health professionals were asked to rate their overall knowledge of HIV and overall knowledge of ART. Ratings of "excellent," "very good," and "good" were grouped together as "sufficient;" 75% of the respondents assessed that their overall knowledge of HIV was sufficient and 40% rated their overall knowledge of ART as sufficient. As shown in Figure 2, there were significant differences in ART knowledge across professions.

Respondents' self-assessment of their ART knowledge was significantly related to training in initiating and monitoring ART. Twenty-two percent of 66 who rated their knowledge of ART as less than good had training on initiating ART; 70% of 199 who rated their knowledge as sufficient had training (p < 0.001). Similarly, 16% of 159 who rated their knowledge of ART as less than good had training on monitoring ART; 54% of 106 who rated their knowledge as sufficient had training (p < 0.001). These measures were significantly related within health professions in seven out of eight comparisons.

## Discussion

Access to ART in Uganda has extended beyond specialized urban clinics to district hospitals and primary care facilities. Although the percentage of HIV patients receiving ART was higher at regional referral hospitals (45%) than at district hospitals (33%) and health centre IVs (17%), the percentages may even out over time if HIV patients transfer their care to accredited facilities closer to their homes.

Uganda's well-developed ART programme was staffed by a broad range of health professionals. Sixty-four percent of professionals who prescribed ART were clinical officers, nurses or midwives. This task-shifting followed the recommendations of experts and may have contributed to extending access to ART.

Training on initiating and monitoring ART had not, however, always kept pace with task shifting. Although the majority of people who prescribed ART had attended training on initiating and monitoring ART, 35% of respondents had not attended training on initiating ART and 49% on monitoring ART. The percentages of people who prescribed ART who had attended no training on monitoring ART were significantly different across health professions: 27% of doctors had no training on monitoring ART, compared to 64% of other clinicians. Similarly, self-assessments of knowledge of ART differed significantly across professions; 7% of doctors who prescribed ART reported their overall knowledge of ART was lower than "good", compared with 48% of other clinicians. The criteria for a health facility to be accredited to provide ART in Uganda included that a minimum number of health professionals were qualified with experience in HIV/AIDS management [20], but the staff of the ART clinics may have changed over time.

This is the first article to document task shifting and training needs across a range of health professionals. Other assessments of a range of health professionals did not document responsibilities for HIV care. Liljestrand reported significant differences in HIV training across professions in the United States; for example, registered nurses had less ART training than doctors, physician assistants and nurse practitioners [27]. The multidisciplinary training needs assessment by the Center for African Family Studies and Regional AIDS Training Network concluded that the two most critical training gaps for doctors, clinical officers and nurses were the same, but it was based on expert opinion rather than self-assessment (Marc Ahmed Okunnu, personal communication, 10 August 2009).

Training needs as measured by previous training and self-assessment of knowledge provided similar results and the measures were significantly related. A review of studies of health professionals in the United States and Europe concluded that the validity of self-assessment of performance was low to moderate, but could be improved with appropriate training [28]. Others recommended assessment based on more objective measures of competence [29]. Our results suggested that self-assessment of overall knowledge of ART was a valid measure of basic ART training needs in a resource-limited setting. Future research on detailed training needs may use diaries in which health professionals note difficult situations during encounters with patients [30].

## Limitations

The health professionals were a convenience sample of those who were at a nationally representative sample of accredited ART clinics on the day of the study. Consequently, the sample overrepresented some types of professionals at some facilities and underrepresented others; doctors were underrepresented at all facilities. The sample may have reflected the time doctors allocated to ART care more accurately than administrative records; higher rates of absenteeism among doctors have also been reported previously [31].

Of the 45 facilities in the nationally representative sample, two remote facilities were replaced with ones that were easier to reach; a team was unable to travel to one facility. To the extent that task shifting was more likely to occur in remote facilities and health professionals in those facilities were less likely to be trained, the sample may have underestimated the extent of task shifting and ART training needs associated with it.

## Conclusion

In a national sample of health facilities that were accredited to provide ART in Uganda, 64% who prescribed ART were clinical officers, nurses or midwives, 41% of whom had not been trained in initiating ART and 64% of whom had not been trained in monitoring ART. Training needs were heterogeneous and differed within professions by the tasks performed. It is important to assess the tasks performed and training needs to allocate training resources appropriately. Training initiatives should be an integral part of the support for task shifting and ensure that ART is used correctly and toxicity or drug resistance do not reverse the successes to date.

## Abbreviations

AIDS: acquired immune deficiency syndrome; ART: antiretroviral therapy; HIV: human immunodeficiency virus; IDI: Infectious Diseases Institute; MOH: Ministry of Health; TASO: The AIDS Support Organization; UNAIDS: Joint United Nations Programme on HIV/AIDS; UNICEF: United Nations Children's Fund; WHO: World Health Organization

## Competing interests

Dr. Keith P.W.J. McAdam states that under his direction, the Infectious Diseases Institute had the following sources of funding, which he does not believe were significant personal conflicts of interest: Pfizer Inc, Exxon Mobile, Gilead, and Becton Dickinson, as well as various public funding agencies.

## Authors' contributions

IML and GS led the design and fieldwork for the training needs assessment. MRW, FL, EN, MS, KPWJM and MAS contributed to the design. RK contributed to the data collection. IML and MRW analysed the data. IML and JHO wrote drafts of the manuscript, and MRW wrote the final version, based on written comments from GS, LMS, RK and MAS. All authors contributed to the final version and approved the text as submitted.

## Supplementary Material

Additional file 1 **Questionnaire for Health Professionals**. Questionnaire for individuals with 6 sections including provision of HIV/AIDS services and training in HIV/AIDS. Click here for file

Additional file 2 **Percentage with previous HIV training, by type of health professional**. Detailed results on 17 topics of HIV training in 4 areas: 1) treatment and care, 2) prevention and counseling, 3) HIV laboratory testing, 4) program management and drug supplies. Click here for file
