# Supplementary material for: Training needs assessment for clinicians at antiretroviral therapy clinics: evidence from a national survey in Uganda
Source: Hum Resour Health. 2009 Aug 23;7:76. doi: 10.1186/1478-4491-7-76 (PMC2752450; doi:10.1186/1478-4491-7-76)
Supplement: Additional file 1 — Questionnaire for Health Professionals. Questionnaire for individuals with 6 sections including provision of HIV/AIDS services and training in HIV/AIDS. [file 1478-4491-7-76-S1.doc]

Questionnaire Number: ____________


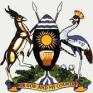
Training needs assessment of Health Workers in ART

**The individual questionnaire**

**Identifier:**

A1. Name of the Health Facility : _________________________________

A2. Name of the District : _________________________________

A3. Name of the County : _________________________________

A4. Date of the interview : Day _________ Month__________ 2006

A5. Checked and edited by : _________________________________

**Introduction:**

The Infectious Diseases Institute (IDI) in collaboration with the Ministry of Health and the support of other stakeholders in HIV and ART training are conducting a training needs assessment. The purpose of this survey is to determine the training needs in various public and private health facilities involved in Antiretroviral Therapy with the aim to provide more effective training and design courses and programmes that will meet the needs of health care providers in the field. We therefore would like to request that you fill this questionnaire to the best of your knowledge. All responses given will be treated with a high degree of **CONFIDENTIALITY.**

Thank you for your assistance.

This questionnaire is to be filled out by **individual health care provider**

**Please note:** Where applicable just CIRCLE the appropriate answer. Thank you!

Time when starting to fill in the questionnaire: _______________hrs

1. **Background**

Q1. Please indicate your age: ________________

Q2. Please indicate your gender: 1. Male 2. Female

Q3. Where do you primarily practice?

1. Region Referral Hospital 2. District Hospital

3. Health centre IV 4. Non Government Organization(NGO)

5. Private clinic 6. Faith Based Organization (FBO)

7. Community Based Organization (CBO) 8. Others, specify __________

Q4. What is your profession? (**Circle all applicable**)

1. Physician / Medical Officer 2. Clinical Officer 3. Nurse

4: Midwife 5. Health assistant 6. Pharmacist

7. Dispenser 8. Counselor 9. Social worker

10. Laboratory technician 11. Laboratory assistant 12. Home visitor

13. Laboratory technologist 14. Other, specify _________________________

**II. PROVISION OF HIV/AIDS CARE SERVICES**

Q5. For how long have you been providing HIV/AIDS care? ______ months _______ years

Q6. In a normal working day, how much time do you spend on any of the following

activities?

|  | **Role** | **In hours** |
| --- | --- | --- |
| 1 | Administration/ supervision |  |
| 2 | Prescribing ART |  |
| 3 | Prescribing other medications |  |
| 4 | Providing basic HIVcare |  |
| 5 | Counseling clients |  |
| 6 | Nursing care to PLWHA |  |
| 7 | Working in the laboratory |  |
| 8 | Training health workers |  |
| 9 | Health education |  |
| 10 | Home visiting |  |
| 11 | Others (specify) …………… |  |

Q7. In your present position, which of the following HIV related activities are you involved in full time (8 hours per day) or part-time (less than 8 hours per day). Please fill in activity 1-24 and tick where applicable

| **Activity** | **Full time** | **Part-time** | **Activity** | **Full time** | **Part-time** |
| --- | --- | --- | --- | --- | --- |
| 1. HIV Counseling |  |  | 13. General outpatient care |  |  |
| 2. HIV Testing and monitoring |  |  | 14. Outpatient care for HIV patients |  |  |
| 3. Home Visiting |  |  | 15. Inpatient Care for HIV/AIDS |  |  |
| 4. PLWHA feeding & Nutrition programs |  |  | 16. Inpatient Care for general patients |  |  |
| 5. Community based Prevention |  |  | 17. PMTCT programme |  |  |
| 6. Reproductive health counseling including A,B, C etc |  |  | 18. ARV Initiation |  |  |
| 7. Condom Distribution |  |  | 19. ARV Follow-up |  |  |
| 8. Home based /Palliative care |  |  | 20. HIV Programme Management |  |  |
| 9. Adolescent Health Programmes |  |  | 21. ARV monitoring and evaluation |  |  |
| 10. Adolescent HIV Care |  |  | 22. ART procurement & supply chain management |  |  |
| 11. School health Programmes |  |  | 23. HIV Policy Issues |  |  |
| 12. Monitoring & Evaluation |  |  | 24. Health information Systems |  |  |

Q8. On average, state the number of patients **you as a health worker** care for as follows:

1. Number of patients that you, **as an individual health worker,** care for on an average clinic day: _______
2. Number of patients with HIV/AIDS that you, **as an individual health worker,** care for on an average clinic day: __________
3. Of the above number in (b), how many patients are on ART __________
4. How many patients do you, **as an individual health worker,** start on ARV’s in one week? _______

Q9. When did you last care for a PLWHA (Person living with HIV/AIDS)?

1. Today 2. Within the last 7 days

3. Within the last month 4. More than a month ago

***If you are not providing ART please skip to Q 14***

Q10. When did you last care for a PLWHA on ART?

1. Today 2. Within the last 7 days

3. Within the last month 4. More than a month ago

Q11. In your health facility what steps do you usually take before starting a patient on ART? (***Please tick if that activity is done before you start a patient on ART*)**

| **Action Item** | **Always done** | **Occasionally done** | **Not done or not available** |
| --- | --- | --- | --- |
| 1. Clinical assessment |  |  |  |
| 2. WHO staging |  |  |  |
| 3. Diagnosis of Opportunistic Infections |  |  |  |
| 4. Treatment of Opportunistic Infections |  |  |  |
| 5. CD4+ count |  |  |  |
| 6. Repeat HIV test |  |  |  |
| 7. HB and FBC (full blood count) |  |  |  |
| 8. Liver function test or renal function tests |  |  |  |
| 9. Identification of a treatment supporter |  |  |  |
| 10. Initial ART counseling session |  |  |  |
| 11. More than one ART counseling session |  |  |  |
| 12. Take detailed address and phone number |  |  |  |
| 13. Home visit |  |  |  |
| 14. HIV Testing of spouses or family members |  |  |  |

Q12. Do you have specific activities which promote adherence to ART (compliance with taking antiretroviral drugs) at your facilities?

1. Yes 2. No

Q13 If yes, which ones? (**Circle what applies**)

1. Counseling 2. Peer support 3. Home visit

4. Other, specify _____________________________________________

**III. TRAINING IN HIV/AIDS**

Q14. Have you ever had any HIV/AIDS training?

1. Yes 2. No ……. ***If no go to Q 16***

Q15. If yes, Please indicate in what subject areas you have been trained?

| **Subject** | **Which year?** | **Training agency/ Institution** | **Length of the course**  In days |
| --- | --- | --- | --- |
| 1. Voluntary or routine counseling and testing |  |  |  |
| 2. Basic HIV care |  |  |  |
| 3. Clinical HIV Care |  |  |  |
| 4. Community Based Care for PLWHA |  |  |  |
| 5. How to start ART |  |  |  |
| 6. How to monitor ART |  |  |  |
| 7. HIV Prevention |  |  |  |
| 8. How to prevent mother to child transmission (PMTCT) |  |  |  |
| 9. How to do research in HIV |  |  |  |
| 10. How to train communities on HIV |  |  |  |
| 11. How to care for children with HIV |  |  |  |
| 12. How to give ART to children |  |  |  |
| 13. How to manage HIV and ART programmes |  |  |  |
| 14. Training of Trainers |  |  |  |
| 15. How to manage drug supplies in ART |  |  |  |
| 16. How to test for HIV |  |  |  |
| 17. Laboratory monitoring in ART |  |  |  |
| 18. Nursing care &HIV Management |  |  |  |
| 19. Monitoring & Evaluation of HIV programmes |  |  |  |
| 20. Breastfeeding, infant feeding in HIV |  |  |  |
| 21. Stigma and discrimination |  |  |  |
| 22. Infection control at health facilities |  |  |  |
| 23. Other, specify: |  |  |  |

Q16. Do you have any responsibility in training other health care workers?

1. Yes 2. No ***If No, go to Q 18***

Q17. If yes, in what subject area(s) do you train others?

_______________________________________________________________________________

_______________________________________________________________________________

Q18. Over all, how would you rate your HIV/AIDS and ART knowledge level?

(**Use:** 1. Excellent 2. Very good 3. Good 4. Fair 5. poor 6. None)

a. **HIV/AIDS**

b. **ART**

Q19. For each of the following areas related to HIV, how would you rate you present knowledge and skill on a scale from 1 – 5 as above.

|  | **HIV related field** | **Please rate here from 1 (excellent) to 5 (poor) your level of skill and knowledge** |
| --- | --- | --- |
| a | Voluntary/Routine counseling and testing |  |
| b | Basic HIV Care |  |
| c | Community Base Care for HIV |  |
| d | Anti-Retroviral Therapy |  |
| e | HIV Prevention |  |
| f | Adherence counseling |  |
| g | Research & HIV |  |
| h | Community educational programmes |  |
| i | Caring for children with HIV and ART |  |
| j | Management of opportunistic infections |  |
| k | Nursing care &HIV Management |  |
| l | PMTCT |  |
| m | Breastfeeding and infant feeding |  |
| n | Counseling issues |  |
| o | Stigma and discrimination |  |
| p | HIV testing |  |
| q | CD 4 testing and other laboratory tests |  |
| r | Infection control in the health care setting |  |
| s | Management of HIV Programmes |  |
| t | Training of Trainers |  |
| u | Monitoring & Evaluation |  |
| v | Logistics management |  |
| w | Supply management in ARV/ drugs |  |
| x | Other ________________________________ |  |

Q20. For which of the following HIV related fields do you perceive a need for training? (***Please tick where applicable***)

|  | **HIV related field** | **Need for training** |
| --- | --- | --- |
| a | Voluntary/Routine counseling and testing |  |
| b | Basic HIV Care |  |
| c | Community Base Care for HIV |  |
| d | Anti-Retroviral Therapy |  |
| e | HIV Prevention |  |
| f | Adherence counseling |  |
| g | Research & HIV |  |
| h | Community educational programmes |  |
| i | Caring for children with HIV and ART |  |
| j | Management of opportunistic infections |  |
| k | Nursing care &HIV Management |  |
| l | PMTCT |  |
| m | Breastfeeding and infant feeding |  |
| n | Counseling issues |  |
| o | Stigma and discrimination |  |
| p | HIV testing |  |
| q | CD 4 testing and other laboratory tests |  |
| r | Infection control in the health care setting |  |
| s | Management of HIV Programmes |  |
| t | Training of Trainers |  |
| u | Monitoring & Evaluation |  |
| v | Logistics management |  |
| w | Supply management in ARV/ drugs |  |
| x | Other ________________________________ |  |

Q22. From the above list (Q21, a-w), choose 4 most important areas where you would like additional training. (**Rank them in order of importance, starting with the most important using the corresponding letter, A to W)**

1. 2. 3. 4.

Q23. Please rate the following modes of training according to your preference:

(***Please Tick Appropriately***)

| **Method of training** | **Very appropriate** | **Appropriate** | **Not appropriate** |
| --- | --- | --- | --- |
| 1. Clinical Case conferences at the H/F |  |  |  |
| 2. Workshops on site |  |  |  |
| 3. Workshops off site |  |  |  |
| 4. Interactive workshops and skill building |  |  |  |
| 5. Free phone consultations with HIV experts |  |  |  |
| 6. Lectures on specific topics on site |  |  |  |
| 7. Conferences on HIV/AIDS or ART (off site) |  |  |  |
| 8. Satellite or videoconferencing |  |  |  |
| 9. Self learning through computer based  facilities (CD-Rom based training) |  |  |  |
| 10. Self learning using video/ DVD based courses |  |  |  |
| 11. Other Types specify  ___________________________________  ___________________________________ |  |  |  |

Q24 a) Would you prefer to train on site or off site?”

1. On site training 2. Off-site training

b) Please explain why?

………………………………………………………………………………………

……….……………………………………………………………………………...

..……………………………………………………………………………………

Q25. Given the nature of your work what would be the maximum number of days that you

could be away from your health facility to do HIV training? _______________

Q26. How far would you be willing to travel for HIV related training?

1. Within the district 2. Anywhere in the country

3. No limitations

Q27. How would you want HIV training to be funded?

1. Fully funded by a donor/ government /Institution
2. Prepared to partially fund myself
3. Prepared to fully fund myself

**IV. BARRIERS TO TRAINING**

Q28. What barriers would make it difficult for you to be trained?

**(*Please indicate by ticking utmost 4 major barriers*)**

| **No.** | **Barriers** | **Tick 4 major ones** |
| --- | --- | --- |
| 1 | Time availability |  |
| 2 | Family reasons |  |
| 3 | Travel costs |  |
| 4 | Tuition fees |  |
| 5 | Lack of support by health facility or agency |  |
| 6 | Work load at the facility |  |
| 7 | Lack of staff for replacements |  |
| 8 | Lack of access to computer |  |
| 9 | Lack of internet access |  |
| 10 | Other reasons  ________________________________ |  |

Q29. What would be needed to overcome the indicated barriers above?

| **Barriers** | **Suggested solution(s)** |
| --- | --- |
| 1. Time availability |  |
| 2. Family reasons |  |
| 3. Travel costs |  |
| 4. Tuition fees |  |
| 5. Lack of support by health facility |  |
| 6. Work load at the facility |  |
| 7. Lack of staff for replacements |  |
| 8. Lack of access to computer |  |
| 9. Lack of internet access |  |
| 10. Other reasons_________________ |  |

**V. ATTENDANCE OF IDI COURSES**

Q30 Do you know about the training programme at the Infectious Diseases Institute(Formerly Academic Alliance)?

1. Yes 2. No ***if no, go to Q 34***

Q31. Have you ever applied for a training course(s) at Infectious Diseases Institute (Formerly Academic Alliance)?

1. Yes 2. No ***if no, go to Q 34***

Q32. If yes, what course(s) did you apply for?

|  | **Course** | **Applied for**  (**Tick**) | **Year of application** |
| --- | --- | --- | --- |
| 1. | HIV/AIDS care core course |  |  |
| 2. | ART programme management |  |  |
| 3. | HIV Research |  |  |
| 4. | Pediatrics/PMTCT |  |  |
| 5. | Training of trainers |  |  |
| 6. | Nursing care and clinic management |  |  |
| 7. | 2 week Nurse and Clinical Officers training |  |  |
| 8. | One week nurses and clinical officers training |  |  |

Q 33 a) Have you ever attended an IDI training course?

1. Yes 2. No if No, go to Q34

b) If so, please indicate which course(s) you attended and when:

|  | **Course** | **Attended**  (Tick) | **Year of attendance** |
| --- | --- | --- | --- |
| 1. | HIV/AIDS care core course |  |  |
| 2. | ART programme management |  |  |
| 3. | HIV Research |  |  |
| 4. | Pediatrics/PMTCT |  |  |
| 5. | Training of trainers |  |  |
| 6. | Nursing care and clinic management |  |  |
| 7. | 2 week Nurse and Clinical Officers training |  |  |
| 8. | One week nurses and clinical officers training |  |  |

**VI . AIDS TREATMENT INFORMATION CENTRE (ATIC)**

Q34. Have ever heard about AIDS Treatment Information Centre (ATIC) at Infectious Diseases Institute?

1. Yes 2. No ……. ***If no, go to end***

Q35. How did you learn about ATIC?

…………………………………………………………………………………………..

Q36. Have you ever consulted them about any complicated HIV/AIDS case or for any HIV information?

1. Yes 2. No ……. ***If no, go to end***

Q37. When was the last time you consulted ATIC? ………………………

Q38. How frequently have you been consulting ATIC? ………………………

***Thank you for your time to complete this questionnaire. Your responses will help Ministry of Health and its partners in training to design trainings that are most needed to improve quality of HIV care in Uganda***

Time when completed the questionnaire _______________________________
