# Supplementary material for: Training needs assessment for clinicians at antiretroviral therapy clinics: evidence from a national survey in Uganda
Source: Hum Resour Health. 2009 Aug 23;7:76. doi: 10.1186/1478-4491-7-76 (PMC2752450; doi:10.1186/1478-4491-7-76)
Supplement: Additional file 2 — Percentage with previous HIV training, by type of health professional. Detailed results on 17 topics of HIV training in 4 areas: 1) treatment and care, 2) prevention and counseling, 3) HIV laboratory testing, 4) program management and drug supplies. [file 1478-4491-7-76-S2.doc]

### Percentage with previous HIV training, by type of health professional

| **Area of training** | **Percentage with previous HIV training** | | | | | **Bivariate analysis** | | |
| --- | --- | --- | --- | --- | --- | --- | --- | --- |
|  | Overall | Doctors | Clinical Officers | Nurses | Mid  wives |  |  |  |
|  | (n=265) | (n=34) | (n=46) | (n=124 | (n=61) | χ^2^ | dF | *p*-value |
| i. Ever been trained | 86 | 82 | 87 | 83 | 93 | 4.11 | 3 | 0.242 |
| ii. Treatment and care |  |  |  |  |  |  |  |  |
| Initiate ART | 43 | 71 | 54 | 40 | 25 | 22.06 | 3 | <0.001^a^ |
| Monitor ART | 32 | 68 | 41 | 26 | 16 | 30.84 | 3 | 0.001^a^ |
| Clinical HIV care | 31 | 56 | 44 | 26 | 18 | 19.57 | 3 | <0.001 |
| Paediatric HIV care | 19 | 32 | 28 | 15 | 13 | 9.54 | 3 | 0.023^a^ |
| Breastfeeding and infant feeding | 22 | 9 | 22 | 17 | 38 | 14.26 | 3 | 0.003^a^ |
| Nursing care & HIV management | 16 | 3 | 13 | 23 | 10 | 10.56 | 3 | 0.014^a^ |
| iii. Prevention and counselling |  |  |  |  |  |  |  |  |
| VCT | 53 | 34 | 44 | 56 | 69 | 13.98 | 3 | 0.003^a^ |
| PMTCT | 52 | 47 | 50 | 44 | 74 | 15.53 | 3 | 0.001^a^ |
| HIV prevention | 37 | 35 | 50 | 37 | 26 | 6.43 | 3 | 0.093 |
| Stigma and discrimination | 14 | 6 | 17 | 15 | 16 | 2.54 | 3 | 0.468 |
| Infection control | 27 | 27 | 30 | 24 | 31 | 1.3 | 3 | 0.729 |
| iv. HIV laboratory testing |  |  |  |  |  |  |  |  |
| Testing for HIV | 17 | 18 | 17 | 15 | 21 | 1.36 | 3 | 0.714 |
| Laboratory monitoring in ART | 5 | 15 | 11 | 3 | 0 | 13.35 | 3 | 0.004^a^ |
| v. Programme management & drug supplies | |  |  |  |  |  |  |  |
| ART supplies | 13 | 27 | 15 | 14 | 2 | 12.81 | 3 | 0.001^a^ |
| HIV research | 2 | 3 | 4 | 2 | 2 | 1.32 | 3 | 0.428^b^ |
| ART/HIV programme management | 11 | 18 | 11 | 7 | 15 | 5.16 | 3 | 0.160^a^ |
| M&E of HIV programmes | 5 | 9 | 11 | 3 | 2 | 6.77 | 3 | 0.099 |
| vi. Training of trainers | 12 | 15 | 15 | 10 | 12 | 0.81 | 3 | 0.469 |

**^a^** – Statistically significant (p<0.05)

b - Used Fisher exact test
